# Determinants of Protective Healthcare Services Awareness among Female Syrian Refugees in Turkey

**DOI:** 10.3390/healthcare10091717

**Published:** 2022-09-08

**Authors:** Mehmet Balcilar, Canan Gulcan

**Affiliations:** 1 Department of Economics, Faculty of Business and Economics, Eastern Mediterranean University, North Cyprus, 10 Via Mersin, Famagusta 99628, Turkey; 2 Department of Economics, OSTIM Technical University, Ankara 06374, Turkey

**Keywords:** Syrian refugees, protective health services awareness, logistic regression, health literacy, socioeconomic status, healthcare use

## Abstract

War-related migration may deprive people of access to a regular healthcare system and cause new diseases to be battled. Since refugee women are more vulnerable to diseases during this period, protective healthcare services awareness is critical for early disease diagnosis. Following the civil war that triggered the migration of millions of Syrians, an extensive survey was undertaken in coordination with the World Health Organization Country Office in Turkey to explore the health status of Syrian refugees in Turkey. Employing the survey data, we aimed to investigate the determinants of the awareness of protective health services (Pap smear test, mammogram, HIV test) among female Syrian refugees. Logit regression analysis was applied in order to investigate the determinants of the awareness of protective health services among the female refugee population. The results revealed a notably low rate of awareness of protective health services among female Syrian refugees. Furthermore, the association of explanatory variables, including socioeconomic factors, healthcare use, and health literacy with the protective health services awareness, was found to be significant.

## 1. Introduction

The Syrian civil conflict, which started in 2011, has forced over 5 million people to flee to other countries. Turkey, hosting about 3.74 million refugees by 2021, is the country with the highest number of Syrian refugees. While 1.4% of those who fled to Turkey as a result of the civil conflict in Syria settled within refugee camps, the other 98.6% settled in cities outside the camps. Refugees, who migrate to other countries due to war or conflict, experience difficulties in accessing a regular healthcare system [1]. Moreover, war-related forced migration may lead to a decline in the quality of life of refugees, new diseases to be battled, and unfavorable living conditions, such as lack of water, electricity, unhygienic conditions, and inadequate nutrition and shelter. Furthermore, the difficulty of monitoring the health status of refugees raises the risk of infectious diseases among this population [2]. Some of the health problems that refugees face include anemia, measles, malaria, respiratory infections, physical violence, and related injuries, sexual abuse, sexually transmitted infections (STIs; e.g., HIV/AIDS), unwanted and risky pregnancies, chronic diseases, depression, anxiety disorders, sleep disorders, and dental problems [3]. Notably, refugee women require additional healthcare as they face special morbidity and mortality risks during this period [4]. However, previous studies have revealed that the percentage of female refugees using healthcare services, particularly for preventative care, is significantly low [4,5]. Besides the risk of many diseases, this low rate of use of healthcare services and preventive health services may also result in undiagnosed cases of cancer, a leading cause of mortality in women, especially breast and cervical cancers [6,7,8,9].

According to the World Health Organization (WHO), cancer is on the list of the top ten diseases that cause death worldwide [10]. Among the different types of cancer, breast and cervical cancers are common diseases affecting women’s health, and their incidences are quite high in this population [11]. For instance, the latest statistics indicate that breast cancer is the leading type of cancer seen in women in comparison to other cancer types. It affected 2.3 million women globally in 2020, with 685,000 deaths [12]. Cervical cancer was found to be the fourth most common cancer in women worldwide; more than 300,000 patients died in 2018 [13]. Additionally, among Syrian women between the ages of 15 and 44 years, cervical cancer is the ninth most common type of cancer [14]. From this perspective, many techniques have been developed for the screening or diagnosis of cervical and breast cancers. Mammography and Pap smear test are the most commonly employed techniques used worldwide [15,16]. There are many country-dependent guidelines for the application of these techniques. For instance, some countries do not suggest that they can be used for patients of certain ages, but some physicians argue that mammograms and/or Pap smears are important for screening starting from younger ages [17]. Many scientific evaluations to date have demonstrated the protective role of these techniques for early diagnosis and treatment options [18]. While the effective use of these techniques is related to the patient’s decision, it is also associated with a broader awareness of protective health services. Although media programs and advertisements both promote and increase awareness about women’s health and women’s diseases, this is less common in some developing and non-developed countries [19,20].

Even though several medical techniques have been developed for the treatment of various types of cancer, the recent trends largely entail protection methods based on knowledge about health-protective services [21]. Similar to other protective health services, many factors have been correlated with the decision to apply mammography or Pap smear test for the routine control of cancer in women [22,23,24]. These services particularly involve coordinated studies between public and health units to encourage people to pursue prospective healthcare for the earlier detection of diseases. Many programs have been used in the last decades very effectively in developed countries leading to the successful treatment of certain cancer types in partial relation to increased awareness of health-protective behaviors [25]. Regarding the high incidence of both cancers, many developed countries have established relevant organizations and policies, such as the Breast and Cervical Cancer Mortality Prevention Act in the United States [26]. Similarly, the European Union has established policies for the prevention of these cancers [27]. These activities primarily aim at the early diagnosis of these diseases as early detection is known to be key for more effective cancer treatment [28,29]. At the same time, these programs also increase public knowledge and, therefore, public awareness. Additionally, the effects of age, marital status, education, income, health literacy, lifestyle, health insurance, access to healthcare, and parental history of cancer have all been investigated in efforts to increase women’s awareness of protective health services for the prevention of cancer and their decisions to use such services [22,30,31,32]. It is well known that age has been linked to cancer development [33]. The recommended age range for routine pap smear and mammography use is between 40 and 50 years old in many developed countries [34,35]. Previous studies investigating the relationship between pap smear and mammogram awareness and age have shown that women’s awareness and concerns regarding mammogram and pap smear tests are related to age [36,37]. Accordingly, the awareness of the protective healthcare services of young women was found to be lower than for older women. The effect of marital status on pap smear and mammogram awareness has been documented by a number of studies [38,39]. Previous research shows that married women are more likely to be aware of pap smear and mammograms compared to singles. Studies clearly show that income and educational background are important motivating variables for the utilization of mammograms and Pap tests [40,41]. Accordingly, women with higher incomes and levels of education are more likely to utilize protective healthcare services than women with lower socioeconomic status. Lack of health insurance was found to be one of the risk factors for postponing healthcare use among immigrants based on previous studies [42]. Accordingly, uninsured immigrants have been found to have lower odds of having had a Pap test and mammogram compared to insured immigrants. Health literacy is explained as “the degree to which individuals have the capacity to obtain, communicate, process, and understand basic health information and services needed to make appropriate health decisions” [43]. Therefore, the utilization and awareness of protective healthcare services, such as cancer screening tests, can be promoted by improving health literacy [44]. 

The transmission of infectious diseases into the host country is linked to migration flows [45]. Infectious diseases, such as respiratory tract infections, diarrheal illnesses, TB, and HIV, continue to be important causes of death and morbidity due to mass immigration caused by war [46]. Since refugees are more likely to develop STIs due to factors related to migration, it is crucial to screen migrants in both transit and host countries to provide alternatives for early diagnosis, prevention and intervention, improve health status, and find infection cases if they exist [45]. Similar to other diseases affecting women, the early diagnosis of STIs is also very important for the health of refugee women, since they experience more health problems than the host population due to migration-related factors such as difficult living conditions, the lack of access to healthcare services, poor health conditions, violence, sexual abuse, and low income [3,47,48]. HIV testing, a protective healthcare service, is a vital starting phase for both diagnosis and treatment of the disease [49]. Although the data related to the prevalence of HIV among Syrian women is limited, it is well known that women are more susceptible to HIV than men with respect to both biological and sociocultural reasons [50]. Other factors leading to HIV among women were reported to include violence against women, low levels of education, lack of knowledge, poverty, migration, and discrimination [50]. Many factors linked to HIV testing, such as marital status, age, pregnancy, socioeconomic status, HIV-related discrimination, number of lifelong partners, health insurance, and quality of healthcare services have been shown by studies in different countries [51,52]. Low levels of health literacy, a concept becoming increasingly critical in public health, may also result in less usage of the preventative health services [53] and poorer health outcomes [54].

As per the researchers’ knowledge, there is no research on this field with such a large sample of the refugee population in Turkey. The aim of this study is to investigate the impacts of the determinants, such as demographics, socioeconomic status (SES), healthcare use, and health literacy, on the awareness of protective health services including mammography, Pap smear, and HIV testing amongst female Syrian refugees. It is considered that this study will contribute to the limited existing literature on the awareness of the protective health services among female Syrian refugees. The ability to understand the effects of these determinants on the protective health services awareness can provide a starting point for policy makers to develop strategies for increasing the knowledge and awareness related to the protective health services of this population. 

The remainder of this paper is organized as follows. Section 2 introduces the materials and methods of the study, including the participants and data collection, measures, and statistical analysis. Section 3 and Section 4 present the empirical results and a discussion, respectively. Finally, the conclusion is identified in Section 5.

## 2. Materials and Methods

### 2.1. Participants and Data Collection

The data, collected by a study in cooperation with the WHO Country Office in Turkey in 2018 and provided to the authors to use for research purpose, was administered to identify and learn more about the health status of Syrian refugees who were not residing in camps [55]. Following two-stage random sampling process is used to identify the sample population: (1) in accordance with the portion of refugees in each province, a sample population for refugees residing outside of camps were computed, (2) 15 provinces (i.e., Adana, Ankara, Bursa, Gaziantep, Hatay, İstanbul, İzmir, Kahramanmaraş, Kayseri, Kilis, Konya, Mardin, Mersin, Osmaniye, Sanliurfa) with the highest Syrian populations were selected in order to comprise a significant proportion (90%) of the overall Syrian refugees [55]. Household selection was carried out by random selection based on the neighborhood lists determined by WHO for each province and the information that included refugee settlements with varying densities obtained from the neighborhood mukhtars of these provinces [55]. Thus, the quantitative survey data included more than 10,000 Syrian respondents (including males, females, and children) from 4068 households from 15 Turkish cities. However, for the aim of this study, male participants and children were excluded and a total of 3442 female Syrian refugees aged 15 and over living in settlements outside of refugee camps were included in the analysis for each response variable. All survey questions were administered as face-to-face interviews and computer-assisted personal interviewing, a method in which the interviewer notes the participants’ answers by entering them into small computer tablets, by trained data collectors. Moreover, all of the field employees used survey materials, such as interviewer and supervisor guidelines, that had been prepared in Arabic, English, and Turkish [55]. 

### 2.2. Measures

The study’s response variables were determined as Pap smear test, mammography, and HIV testing awareness. The outcome variables were assessed by asking participants to answer “yes” or “no” to the following closed-ended questions: (1) “Are you aware of Pap smear screening?” (2) “Are you aware of mammography screening?” (3) “Are you aware of HIV testing?”. The reference category for each response variable was determined as “no.” Based on the findings from other studies on the awareness of protective health services, we determined the control variables as follows: (a) characteristics of the refugees, such as age, marital status, pre-migration residency, divided into two categories including Aleppo and others (i.e., Daraa, Deir ez-Zor, Hama, Al-Hasakah, Homs, Idlib, Quneitra, Lattakia, Al-Raqqah, As-Suwayda, Rif Dimashq, Tartus, Damascus), the number of years spent in Turkey, and the number of pregnancies; (b) socioeconomic status, including education (illiterate, literate, primary/secondary school graduates, high school, university/post graduates), pre-migration and post-migration income (in log), pre-migration and post-migration employment status; (c) healthcare use (having health insurance and using health services); and (d) health literacy. In brief, the categorical variables’ reference groups were organized as follows: (1) marital status: single, (2) pre-migration residency: other, (3) education: illiterate, (4) pre-migration and post-migration employment status: unemployed, (5) having health insurance: no, and (6) use of health services: no. Nine of 13 questions from the Eastern-Middle Eastern Adult Health Literacy Screening instrument (EMAHL13), scored on a 5-point Likert-type scale (i.e., “never,” “rarely,” “sometimes,” “most of the time,” and “always”), were employed to measure the health literacy of the respondents [56].

### 2.3. Statistical Analysis

All statistical analyses were performed using IBM SPSS Statistics version 21.0 (Armonk, New York, NY, USA). Statistical significance tests (chi-squared tests for categorical variables and t-tests for continuous variables) were applied to observe the differences among the variables associated with protective health services. Multiple logistic regression modeling, a technique commonly used to determine relationships between explanatory variables and response variables with two or more categories, was applied separately for each response variable to investigate the potential predictors associated with awareness of protective health services. The results of the analyses were presented with coefficients and odds ratios (ORs) estimated with confidence intervals of 95%.

## 3. Results

Table 1 summarizes the descriptive statistics of all of the factors that were utilized in this research. Accordingly, the average age of the participants was 29.53; 19.7% of the respondents were single and 80.3% were married. The average number of years spent in Turkey following migration was approximately 3.4 years, and the mean number of pregnancies was 2.57.

Refugees from Aleppo constituted the highest proportion of the sample population in the analysis at 61%. The majority of the participants (57.6%) had at least a primary/middle school diploma. The pre-migration and post-migration employment status were reported as unemployed at rates of 96.6% and 95.9%, respectively. The average log incomes before and after migration were 8.45 and 6.86, respectively. While 90.4% of participants indicated having no health insurance, 67.3% did report using health services. Finally, the mean EMAHL13 score of health literacy was 17.7 among female refugees. 

Considering the logistic regression analyses applied for the odds of the specified indicators of awareness of protective health services, age is not a significant determinant of Pap smear (Table 2). On the other hand, it does make a substantial contribution to mammography awareness (OR = 1.027, 95% CI [1.006, 1.048]) (Table 3). Besides, age has been found to be insignificant on HIV testing awareness (Table 4). As can be seen in Table 4, the number of pregnancies and the number of years spent in Turkey were found to be positively associated only with HIV testing awareness among these refugees. Accordingly, for every one-unit increase in the number of pregnancies and the number of years spent in Turkey, the log odds of HIV testing awareness increase by factors of 1.089 (95% CI [1.014, 1.169]) and 1.175 (95% CI [1.039, 1.329]), respectively.

The findings suggest that having a high school education or university/postgraduate degree is linked to a higher likelihood of being aware of protective health services, such as mammography and HIV testing (Table 3 and Table 4). There is, however, no link between Pap smear test awareness and education (Table 2). It can be seen that the pre-migration incomes of the refugee women are highly positively associated with awareness of Pap smear screening (OR = 1.235, 95% CI [1.098, 1.389]) (Table 2), while post-migration incomes are negatively associated with Pap smear (OR = 0.776, 95% CI [0.621, 0.969]) and mammography awareness (OR = 0.784, 95% CI [0.639, 0.962]) (Table 2 and Table 3).

Notably, one can see that the effect of having health insurance on awareness of protective health services, i.e., Pap smears and mammography, is quite significant. Accordingly, refugee women who have health insurance are more likely to be aware of Pap smear test and mammography screening by factors of 3.1 (95% CI [1.828, 5.313]) and 2.6 (95% CI [1.512, 4.496]), respectively (Table 2 and Table 3). The odds of HIV testing awareness, on the other hand, are 2.12 (95% CI [1.325, 3.408]) times higher for those who use health services compared to those who do not. Overall, our results reveal that health literacy levels are highly positively associated with awareness of Pap smear screening (Table 2). For every one-unit increase in the level of health literacy, the odds of the awareness of Pap smear screening increase by a factor of 1.05 (95% CI [1.025, 1.065]). There is, on the other hand, no association between the awareness of mammography or HIV testing and the health literacy of these female Syrian refugees. Additionally, pre-migration residency, marital status, and pre-migration and post-migration employment status were not found to be associated with the awareness of Pap smears, mammography, or HIV testing.

Table 2, Table 3 and Table 4 indicate that about 8.1%, 4.5%, and 4.5% of the variance in the outcome variables (Pap smear, mammograms, and HIV testing) are explained according to McFadden R-squared, respectively.

## 4. Discussion

This study aimed to identify determinants of the awareness of protective health services related to cancers specific to women (e.g., breast and cervical cancers) and STIs (e.g., HIV) among female Syrian refugees. In general, awareness of all protective health services was found to be quite low among the sample population. This outcome implies that these women were also unaware of the considered screening and diagnosis techniques in their regular pre-migration lives. The findings that we found related to the low rate of awareness of protective health services among Syrian refugees have similarities with the outcomes of some previous studies conducted on Arab women. For instance, a study conducted on the employment of these screening tests among women in Jordan reported that the prevalence of Pap smear cancer screening and mammogram was 15.3%, and 8.7%, respectively [57]. Moreover, a study based on attitudes toward cervical cancer screening of Muslim women immigrants living in the U.S. has also shown a lower rate of cancer screening activities among this population [58]. Some studies further questioned this low prevalence and suggested the significance of religion and beliefs to be critical in both the awareness and usage of screening techniques [58]. Therefore, the low percentage of awareness of protective health services among female Syrian refugees in the present study is comparable to the findings of previous research. 

Age has previously been shown to be one of the factors associated with awareness of screening tests for the diseases considered in earlier studies [59]. We have shown here, however, that age is only significant for mammography screening awareness. Accordingly, an increase in age leads to an increase in the awareness of mammogram screening. The development of breast cancer in women is also age-dependent [33], and many developed countries’ health policies suggest the routine use of Pap smears and mammograms starting from the age of about 40–50 years unless there is genetic susceptibility to the diseases in question [34,35]. We also found positive and significant relationships between both the number of years spent in Turkey and the number of pregnancies and awareness of HIV testing. While in the United States and European countries, prenatal HIV testing is considered a regular part of healthcare services, rates of HIV testing among pregnant women are still low in many other countries [60]. Since women are tested for HIV in the early months of pregnancy in Turkey, this routine testing and increased time spent in Turkey may have particularly increased HIV awareness among pregnant Syrian refugees.

The effects of education and pre-migration income were also found to be positive for the awareness of protective health services. It was clearly shown that illiterate female Syrian refugees have less awareness in comparison to others with higher education levels. Moreover, refugees who had higher levels of pre-migration income were more likely to be aware of mammography as a screening technique. Parallel to our findings, many studies have confirmed that low income and lower levels of education are linked to lower levels of cancer education among patients [61,62,63]. Moreover, previous studies have also shown that women with higher levels of education and income have higher awareness of preventive health services than those with lower levels of education and income [40,41]. Having health insurance and using health services are factors that have been determined to be significant contributors to the awareness of protective health services. Since it is well known that refugees may have difficulties in accessing healthcare services due to various uncertainties, security concerns, language problems, cultural differences, and financial issues, it is critical to improve these women’s access to healthcare [4]. Health literacy is a highly significant indicator of awareness of pap smear. Accordingly, it was revealed here that higher levels of health literacy correlated with higher levels of awareness of Pap smear screening among female Syrian refugees. This is an expected finding since many studies to date have depicted positive relationships between health literacy and awareness of cancer screening tests [64,65,66,67]. Therefore, it is crucial to provide educational programs that will help to improve the health literacy of this population to increase awareness of preventive healthcare services. 

Previous studies on the awareness of screening for certain cancer types have already identified lower frequencies among Muslim women [68]. Concerning awareness of HIV testing, it is unusual for women in Arab societies to have more than one sexual partner, and that might result in lower rates of HIV prevention awareness [69]. Based on the present results, it is difficult to make an overall generalization of this population’s awareness of protective health services, since the geographical and environmental factors at play before and after the Syrian civil war are quite different. This topic warrants further investigation to underline the effects of social, geographical, and cultural factors on awareness of protective health services. On the other hand, awareness of screening tests among Turkish women is quite high, comparable to the rates found in other developed or developing countries [70]. Considering the fact that Syrian and Turkish women live together today within different regions of Turkey, educational programs must be organized by different Turkish institutions to increase the awareness of protective health services and promote screening tests among Syrian refugees. 

## 5. Conclusions

To our knowledge, this research has been the first of its kind in terms of investigating the awareness of protective health services among such a large sample of female Syrian refugees living in Turkey. In comparison to similar studies on Syrian refugees who have migrated to countries other than Turkey, this study is also the most comprehensive one to date concerning the number of participants included. This study, by investigating the factors affecting the awareness of preventive health services among Syrian women who immigrated to Turkey after the war, might give an idea to the authorities for strategies that can be developed to increase awareness of these services and promote better health outcomes. 

A detailed analysis of the data in this study may lead to a number of recommendations. Because many of the variables examined herein were shown to be the factors that affect female Syrian refugees’ awareness of preventive health services, policymakers should conduct educational steps to enhance awareness of preventive health services and motivate Syrian refugees to participate in screening tests. Since inadequate health literacy might lead to lack of knowledge about health problems, lack of health preventive awareness, and an increase in hospitalization, necessary steps should be taken by policy makers to evaluate the level of health literacy among female refugees. Moreover, advertising campaigns should be made available to increase the awareness of preventive health services in this population and to make them understand the importance of early diagnosis of diseases.

The results of this analysis may be extended for further research. Firstly, further statistics routinely monitoring the change of the awareness of female Syrian refugees against these screening tests might be ideal for future assessments. Secondly, effective communication between refugees and healthcare professionals is seriously affected by language barriers. Therefore, language and communication barriers that refugees might experience in the host country should be addressed in the analysis. Lastly, it is noteworthy to highlight that this study should be viewed as preliminary and that further research is required to analyze more factors that may be linked to awareness of protective health services among the refugee population.

## Figures and Tables

**Table 1:** Descriptive statistics of the sample population.

Explanatory Variables	(N)	%	Mean	Std. Dev.
Characteristics of the sample population				
Age	3442		29.53	9.244
Marital Status married single	3442	80.3 19.7	0.20	0.398
Years in Turkey	3302		3.39	1.430
Pre-migration residency Aleppo Other	3442	61.0 34.9	0.36	0.481
Number of pregnancies	3301		2.57	2.231
Socioeconomic status				
Education illiterate literate primary/secondary school high school university/post-graduate	3442	24.5 8.70 57.6 7.10 2.20	2.46	1.005
Pre-migration income (in the log)	3442		8.45	2.01
Post-migration income (in the log)	3442		6.86	0.99
Pre-migration employment status employed unemployed	3436	3.20 96.6	0.97	0.17
Post-migration employment status employed unemployed	3436	3.90 95.9	0.96	0.19
Health-care use				
Health insurance yes no	3302	5.50 90.4	0.94	0.233
Health services use yes no	3302	67.3 28.7	0.30	0.458
Health Literacy	3302		17.7	9.445
Response Variables:				
Awareness of pap-smear screening yes no	3301	4.0 91.9	0.96	0.200
Awareness of mammography screening yes no	3301	4.4 91.5	0.95	0.210
Awareness of HIV test yes no	3302	4.9 91.1	0.95	0.220

**Table 2:** Logistic regression estimates.

Response Variable (Pap-Smear) ^h^	β	Prob.	OR (CI 95%)
Demographics			
Age	0.020	0.076	1.020 (0.998–1.043)
Marital Status ^a^ married	0.307	0.252	1.360 (0.804–2.300)
Number of pregnancies	−0.086	0.097	0.918 (0.829–1.016)
Years in Turkey	0.120	0.080	1.127 (0.986–1.290)
Pre-migration residency ^b^ Aleppo	−0.107	0.624	0.899 (0.586–1.378)
Socio-economic status			
Education ^c^ university/post-grad high school primary/secondary school literate	0.773 −0.127 −0.019 0.154	0.126 0.754 0.940 0.694	2.167 (0.805–5.837) 0.880 (0.397–1.952) 0.981 (0.597–1.611) 1.166 (0.542–2.509)
Pre-migration income (in log)	0.211 ***	0.000	1.235 (1.098–1.389)
Post-migration income (in log)	−0.254 *	0.025	0.776 (0.621–0.969)
Pre-migration employment status ^d^ employed	0.566	0.279	1.760 (0.633–4.897)
Post-migration employment status ^e^ employed	−0.740	0.253	0.477 (0.134–1.695)
Health-care use			
Health insurance ^f^ yes	1.137 ***	0.000	3.116 (1.828–5.313)
Health services use ^g^ yes	0.258	0.262	1.295 (0.824–2.034)
Health Literacy	0.044 ***	0.000	1.045 (1.025–1.065)
Pseudo R-squared	0.081		

Note: Reference categories are (^a^) single, (^b^) other, (^c^) illiterate, (^d,e^) unemployed, (^f,g,h^) no. *** *p* < 0.001; and * *p* < 0.05. EMAHL13 questionnaire consisted of 9 items and the value for Cronbach’s Alpha for the questionnaire was found to be α = 0.97 in this study.

**Table 3:** Logistic regression estimates.

Dependent Variable (Mammography) ^h^	β	Prob.	OR (CI 95%)
Demographics			
Age	0.027 *	0.010	1.027 (1.006–1.048)
Marital Status ^a^ married	0.369	0.162	1.446 (0.863–2.423)
Number of pregnancies	0.000	0.996	1.000 (0.921–1.085)
Years in Turkey	0.015	0.814	1.015 (0.896–1.151)
Pre-migration residency ^b^ Aleppo	0.232	0.266	1.261 (0.838–1.895)
Socio-economic status			
Education ^c^ university/post-grad high school primary/secondary school literate	1.442 ** 0.749 * 0.367 0.001	0.003 0.035 0.134 0.999	4.228 (1.619–11.04) 2.114 (1.053–4.244) 1.444 (.893–2.334) 1.001 (.456–2.195)
Pre-migration income (in log)	0.093	0.103	1.097 (0.981–1.227)
Post-migration income (in log)	−0.244 *	0.020	0.784 (0.639–0.962)
Pre-migration employment status ^d^ employed	0.503	0.295	1.654 (0.645–4.243)
Post-migration employment status ^e^ employed	−0.426	0.458	0.653 (0.212–2.009)
Health-care use			
Health insurance ^f^ yes	0.958 **	0.001	2.607 (1.512–4.496)
Health services use ^g^ yes	0.221	0.294	1.247 (0.825–1.884)
Health Literacy	0.006	0.530	1.006 (0.987–1.025)
Pseudo R-squared	0.045		

Note: Reference categories are (^a^) single, (^b^) other, (^c^) illiterate, (^d,e^) unemployed, (^f,g,h^) no. ** *p* < 0.01; and * *p* < 0.05. EMAHL13 questionnaire consisted of 9 items and the value for Cronbach’s Alpha for the questionnaire was found to be α = 0.97 in this study.

**Table 4:** Logistic regression estimates.

Dependent Variable (HIV Test) ^h^	β	Prob	OR (CI 95%)
Demographics:			
Age	0.012	0.271	1.012 (0.991–1.033)
Marital Status ^a^ married	0.238	0.354	1.268 (0.767–2.096)
Number of pregnancies	0.085 *	0.019	1.089 (1.014–1.169)
Years in Turkey	0.162 *	0.010	1.175 (1.039–1.329)
Pre-migration residency ^b^ Aleppo	−0.116	0.547	0.890 (0.610–1.300)
Socio-economic status:			
Education ^c^ university/post-grad. high school primary/secondary school literate	0.976 0.915 ** 0.272 −0.236	0.066 0.006 0.263 0.569	2.655 (0.937–7.521) 2.498 (1.294–4.820) 1.313 (0.815–2.116) 0.789 (0.350–1.783)
Pre-migration income (in log)	−0.033	0.563	0.968 (0.867–1.081)
Post-migration income (in log)	0.151	0.143	1.162 (0.950–1.422)
Pre-migration employment status ^d^ employed	0.606	0.202	1.833 (0.723–4.646)
Post-migration employment status ^e^ employed	−0.539	0.344	0.583 (0.191–1.780)
Health-care use:			
Health insurance ^f^ yes	0.037	0.922	1.037 (0.498–2.159)
Health services use ^g^ yes	0.754 **	0.002	2.125 (1.325–3.408)
Health Literacy	0.004	0.684	1.004 (0.985–1.023)
Pseudo R-squared	0.045		

Note: Reference categories are (^a^) single, (^b^) other, (^c^) illiterate, (^d,e^) unemployed, (^f,g,h^) no. ** *p* < 0.01; and * *p* < 0.05. EMAHL13 questionnaire consisted of 9 items and the value for Cronbach’s Alpha for the questionnaire was found to be α = 0.97 in this study.

## Data Availability

Data was obtained from external parties and authors do not have redistribution rights.

## References

[1] Sheikh-Mohammed M., MacIntyre C.R., Wood N.J., Leask J., Isaacs D. (2006). Barriers to access to health care for newly resettled sub-Saharan refugees in Australia. Med. J. Aust..

[2] Altintas H.K., Karadag O. (2010). Refugees and health. Turk Silahlı Kuvvetleri Koruyucu Hekim. Bul..

[3] Yavuz Ö. (2015). Türkiye’ki Suriyeli Mültecilere Yapılan Sağlık Yardımların Yasal ve Etik Temelleri/The Legal and Ethical Foundations of Health Assistances to Syrian Refugees in Turkey. Mustafa Kemal Üniversitesi Sos. Bilimler Enstitüsü Derg..

[4] Erenoğlu R., Sözbir Ş.Y. (2020). The effect of health education given to Syrian refugee women in their own language on awareness of breast and cervical cancer, in turkey: A randomized controlled trial. J. Cancer Educ..

[5] Barın H. (2015). Syrian women in Turkey: Social struggles and solution proposals. GÖÇ.

[6] Orhan O., Gündoğar S.S. (2015). Effects of the Syrian refugees on Turkey. Orsam. Rep..

[7] Zencir M., Davas A. (2014). Syrian Refugees and Health Services Report.

[8] Bebis H., Reis N., Yavan T., Bayrak D., Unal A., Bodur S. (2012). Effect of health education about cervical cancer and papanicolaou testing on the behavior, knowledge, and beliefs of Turkish women. Int. J. Gynecol. Cancer.

[9] Shah S.M., Ayash C., Pharaon N.A., Gany F.M. (2008). Arab American immigrants in New York: Health care and cancer knowledge, attitudes, and beliefs. J. Immigr. Minor. Health.

[10] World Health Organization https://www.who.int/news-room/fact-sheets/detail/the-top-10-causes-of-death.

[11] Ramaswami R., Paulino E., Barrichello A., Nogueira-Rodrigues A., Bukowski A., Louis J.S., Goss P.E. (2018). Disparities in breast, lung, and cervical cancer trials worldwide. J. Glob. Oncol..

[12] World Health Organization https://www.who.int/news-room/fact-sheets/detail/breast-cancer.

[13] Moudatsou M., Vouyiouka P., Karagianni-Hatziskou E., Rovithis M., Stavropoulou A., Koukouli S. (2022). Knowledge and Use of Cervical Cancer Prevention Services among Social Work and Nursing University Students. Healthcare.

[14] HPV Information Centre. https://hpvcentre.net/statistics/reports/SYR.pdf.

[15] Phaswana-Mafuya N., Peltzer K. (2018). Breast and cervical cancer screening prevalence and associated factors among women in the South African general population. Asian Pac. J. Cancer Prev..

[16] Winters S., Martin C., Murphy D., Shokar N.K. (2017). Breast cancer epidemiology, prevention, and screening. Prog. Mol. Biol. Transl. Sci..

[17] Nguyen T.T., McPhee S.J., Nguyen T., Lam T., Mock J. (2002). Predictors of cervical Pap smear screening awareness, intention, and receipt among Vietnamese-American women. Am. J. Prev. Med..

[18] Yip C.H., Taib N.A., Song C.V., Singh R.P., Agarwal G. (2018). Early diagnosis of breast cancer in the absence of population-based mammographic screening in Asia. Curr. Breast Cancer Rep..

[19] Metwally O., Blumberg S., Ladabaum U., Sinha S.R. (2017). Using social media to characterize public sentiment toward medical interventions commonly used for cancer screening: An observational study. J. Med. Internet Res..

[20] Otero-Sabogal R., Stewart S., Sabogal F., Brown B.A., Pérez-Stable E.J. (2003). Access and attitudinal factors related to breast and cervical cancer rescreening: Why are Latinas still underscreened?. Health Educ. Behav..

[21] Amuta A.O., Mkuu R.S., Jacobs W., Ejembi A.Z. (2018). Influence of cancer worry on four cancer related health protective behaviors among a nationally representative sample: Implications for health promotion efforts. J. Cancer Educ..

[22] Anwar S.L., Tampubolon G., Van Hemelrijck M., Hutajulu S.H., Watkins J., Wulaningsih W. (2018). Determinants of cancer screening awareness and participation among Indonesian women. BMC Cancer.

[23] Barbosa Y.C., Oliveira A.G.C., Rabêlo P.P.C., Silva F.D.S., Santos A.M.D. (2019). Factors associated with lack of mammography: National Health Survey, 2013. Rev. Bras. Epidemiol..

[24] Tavasoli S.M., Kane E., Chiarelli A.M., Kupets R. (2018). Women’s behaviors toward mammogram and Pap test: Opportunities to increase cervical cancer screening participation rates among older women. Women’s Health Issues.

[25] Li J., Shao Z. (2015). Mammography screening in less developed countries. Springerplus.

[26] Rim S.H., Allaire B.T., Ekwueme D.U., Miller J.W., Subramanian S., Hall I.J., Hoerger T.J. (2019). Cost-effectiveness of breast cancer screening in the National Breast and Cervical Cancer Early Detection Program. Cancer Causes Control.

[27] Gianino M.M., Lenzi J., Bonaudo M., Fantini M.P., Siliquini R., Ricciardi W., Damiani G. (2018). Organized screening programmes for breast and cervical cancer in 17 EU countries: Trajectories of attendance rates. BMC Public Health.

[28] Lee N.C., Wong F.L., Jamison P.M., Jones S.F., Galaska L., Brady K.T., Stokes-Townsend G.A. (2014). Implementation of the national breast and cervical cancer early detection program: The beginning. Cancer.

[29] Ott J.J., Ullrich A., Miller A.B. (2009). The importance of early symptom recognition in the context of early detection and cancer survival. Eur. J. Cancer.

[30] Ayinde O.A., Ogunbode O.O., Adebayo O.J. (2005). Determinants of cervical cancer knowledge and the utilisation of screening among a Nigerian female population. Trop. J. Obstet. Gynaecol..

[31] Tur-Sinai A., Shahrabani S. (2020). Determinants of women’s decision to undergo early mammography: A survey study. Nurs. Health Sci..

[32] Lee S.Y.D., Tsai T.I., Tsai Y.W., Kuo K.N. (2012). Health literacy and women’s health-related behaviors in Taiwan. Health Educ. Behav..

[33] Mandelblatt J., Andrews H., Kerner J., Zauber A., Burnett W. (1991). Determinants of late-stage diagnosis of breast and cervical cancer: The impact of age, race, social class, and hospital type. Am. J. Public Health.

[34] Liang W., Shediac-Rizkallah M.C., Celentano D.D., Rohde C. (1999). A population-based study of age and gender differences in patterns of health-related behaviors. Am. J. Prev. Med..

[35] Phillips K.A., Kerlikowske K., Baker L.C., Chang S.W., Brown M.L. (1998). Factors associated with women’s adherence to mammography screening guidelines. Health Serv. Res..

[36] Ideström M., Milsom I., Andersson-Ellström A. (2002). Knowledge and attitudes about the pap-smear screening program: A population-based study of women aged 20–59 years. Acta Obstet. Gynecol. Scand..

[37] Elobaid Y.E., Aw T.C., Grivna M., Nagelkerke N. (2014). Breast Cancer Screening Awareness, Knowledge, and Practice among Arab Women in the United Arab Emirates: A Cross-Sectional Survey. PLoS ONE.

[38] Obiechina N.J.A., Mbamara S.U. (2009). Knowledge attitude and practice of cervical cancer screening among sexually active women in Onitsha, southeast Nigeria. Niger. J. Med..

[39] La Frinere-Sandoval Q.N.N.B., Cubbin C., DiNitto D.M. (2022). Perceived neighborhood social cohesion and cervical and breast cancer screening utilization among US-born and immigrant women. AIMS Public Health.

[40] Garcia R.Z., Carvajal S.C., Wilkinson A.V., Thompson P.A., Nodora J.N., Komenaka I.K., Brewster A., Cruz G.I., Wertheim B.C., Bondy M.L. (2012). Factors that influence mammography use and breast cancer detection among Mexican-American and African-Americans women. Cancer Causes Control.

[41] Rosenberg L., Wise L.A., Palmer J.R., Horton N.J., Adams-Campbell L.L. (2005). A multilevel study of socioeconomic predictors of regular mammography use among African-American women. Cancer Epidemiol. Biomark. Prev..

[42] Carrasquillo O., Pati S. (2004). The role of health insurance on Pap smear and mammography utilization by immigrants living in the United States. Prev. Med..

[43] Institute of Medicine of the National Acadamies (2004). Health Literacy: A Prescription to End Confusion.

[44] Han H.-R., Song Y., Kim M., Hedlin H.K., Kim K., Ben Lee H., Roter D. (2017). Breast and Cervical Cancer Screening Literacy Among Korean American Women: A Community Health Worker–Led Intervention. Am. J. Public Health.

[45] Napoli C., Dente M.G., Kärki T., Riccardo F., Rossi P., Declich S., Network for the Control of Cross-Border Health Threats in the Mediterranean Basin and Black Sea (2015). Screening for infectious diseases among newly arrived migrants: Experiences and practices in non-EU countries of the Mediterranean Basin and Black Sea. Int. J. Environ. Res. Public Health.

[46] Hussein N.R., Abdullah I.M., Younus O.M., Taher A.M., Salim A.A., Shahab F.I. (2017). Prevalence of HBV, HCV and HIV infections among Syrian refugees in Kurdistan region, Iraq. Int. J. Infect..

[47] UK Refugee Council (2005). A Study of Asylum Seekers with Special Needs.

[48] Frantz E. (2003). Report on the Situation of Refugees in Turkey: Findings of a Five-Week Exploratory Study December 2002–January 2003.

[49] Musumari P.M., Techasrivichien T., Srithanaviboonchai K., Tangmunkongvorakul A., Ono-Kihara M., Kihara M. (2020). Factors associated with HIV testing and intention to test for HIV among the general population of Nonthaburi Province, Thailand. PLoS ONE.

[50] Türmen T. (2003). Gender and HIV/aids. Int. J. Gynecol. Obstet..

[51] Lakhe N., Diallo K., Ndour C. (2019). Coverage and Associated Factors for HIV Screening in Senegal: Further Analysis of the 2017 Deographic and Health Survey.

[52] Sood N., Wagner Z., Wu Y. (2015). The impact of insurance on HIV testing. Am. J. Health Econ..

[53] Brabers A.E., Rademakers J.J., Groenewegen P.P., Van Dijk L., De Jong J.D. (2017). What role does health literacy play in patients’ involvement in medical decision-making?. PLoS ONE.

[54] Kilfoyle K.A., Vitko M., O’Conor R., Bailey S.C. (2016). Health literacy and Women’s reproductive health: A systematic review. J. Womens Health.

[55] Mipatrini D., Balcılar M., Dembech M., Ergüder T., Ursu P. (2019). Survey on the Health Status, Services Utilization and Determinants of Health: Syrian Refugee Population in Turkey (No. WHO/EURO: 2019-3472-43231-60591).

[56] Nair S.C., Satish K.P., Sreedharan J., Ibrahim H. (2016). Assessing health literacy in the eastern and middle-eastern cultures. BMC Public Health.

[57] Pengpid S., Peltzer K., Zhang C. (2021). Uptake and correlates of cervical and breast cancer screening among women in Jordan: National results of the 2017–2018 Population and Family Health Survey. Gend. Behav..

[58] Matin M., LeBaron S. (2004). Attitudes toward cervical cancer screening among Muslim women: A pilot study. Women Health.

[59] Tekkel M., Veideman T., Baburin A., Rahu M. (2007). Use of mammography and Pap smear in Estonia, a country without organized cancer screening. Int. J. Public Health..

[60] Ben-Natan M., Hazanov Y. (2015). Women’s willingness to be tested for human immunodeficiency virus during pregnancy: A review. World, J. Virol..

[61] Al-Dubai S.A., Qureshi A.M., Saif-Ali R., Ganasegeran K., Alwan M.R., Hadi J.I. (2011). Awareness and knowledge of breast cancer and mammography among a group of Malaysian women in Shah Alam. Asian Pac. J. Cancer Prev..

[62] Macleod U., Mitchell E.D., Burgess C., Macdonald S., Ramirez A.J. (2009). Risk factors for delayed presentation and referral of symptomatic cancer: Evidence for common cancers. Br. J. Cancer..

[63] Al Qadire M., Aljezawi M.E., Al-Shdayfat N. (2019). Cancer awareness and barriers to seeking medical help among Syrian refugees in Jordan: A baseline study. J. Cancer Educ..

[64] Guerra C.E., Krumholz M., Shea J.A. (2005). Literacy and knowledge, attitudes and behavior about mammography in Latinas. J. Health Care Poor Underserved.

[65] Davis T.C., Williams M.V., Marin E., Parker R.M., Glass J. (2002). Health literacy and cancer communication. CA Cancer J. Clin..

[66] Lindau S.T., Tomori C., Lyons T., Langseth L., Bennett C.L., Garcia P. (2002). The association of health literacy with cervical cancer prevention knowledge and health behaviors in a multiethnic cohort of women. Am. J. Obstet. Gynecol..

[67] Lindau S.T., Basu A., Leitsch S.A. (2006). Health literacy as a predictor of follow-up after an abnormal pap smear. J. Gen. Intern. Med..

[68] Azaiza F., Cohen M. (2006). Health beliefs and rates of breast cancer screening among Arab women. J. Womens Health.

[69] Barakat H. (1993). The Arab World: Society, Culture, and State.

[70] Bayçelebi G., AYDIN F., Gökosmanoğlu F., Tat T.S., VARIM C. (2015). Trabzon’da kanser tarama testleri farkındalığı. J. Biol. Rhythm..

